# Graphical comparisons of relative disease burden across multiple risk factors

**DOI:** 10.1186/s12874-019-0827-4

**Published:** 2019-09-11

**Authors:** John Ferguson, Neil O’Leary, Fabrizio Maturo, Salim Yusuf, Martin O’Donnell

**Affiliations:** 1 Health Research Board Clinical Research Facility, Department of Medicine, NUI Galway, Galway, Ireland; 2 Population Health Research Institute, McMaster University and Hamilton Health Sciences, Hamilton, ON Canada

## Abstract

**Background:**

Population attributable fractions (PAF) measure the proportion of disease prevalence that would be avoided in a hypothetical population, similar to the population of interest, but where a particular risk factor is eliminated. They are extensively used in epidemiology to quantify and compare disease burden due to various risk factors, and directly influence public policy regarding possible health interventions. In contrast to individual specific metrics such as relative risks and odds ratios, attributable fractions depend jointly on both risk factor prevalence and relative risk. The relative contributions of these two components is important, and usually needs to be presented in summary tables that are presented together with the attributable fraction calculation. However, representing PAF in an accessible graphical format, that captures both prevalence and relative risk, may assist interpretation.

**Methods:**

Taylor-series approximations to PAF in terms of risk factor prevalence and log-odds ratio are derived that facilitate simultaneous representation of PAF, risk factor prevalence and risk-factor/disease log-odds ratios on a single co-ordinate axis. Methods are developed for binary, multi-category and continuous exposure variables.

**Results:**

The methods are demonstrated using INTERSTROKE, a large international case control dataset focused on risk factors for stroke.

**Conclusions:**

The described methods could be used as a complement to tables summarizing prevalence, odds ratios and PAF, and may convey the same information in a more intuitive and visually appealing manner. The suggested nomogram can also be used to visually estimate the effects of health interventions which only partially reduce risk factor prevalence. Finally, in the binary risk factor case, the approximations can also be used to quickly convert logistic regression coefficients for a risk factor into approximate PAFs.

**Electronic supplementary material:**

The online version of this article (10.1186/s12874-019-0827-4) contains supplementary material, which is available to authorized users.

## Background

Attributable fractions [1] have become a common way of measuring the disease burden attributable to a risk factor on a population level. More precisely, they measure that portion of disease prevalence which would be avoided in a hypothetical population where a particular risk factor was entirely eliminated, but is otherwise identical to the population of interest. Depending on the author, this quantity is referred to variously as a population attributable fraction (PAF), population attributable risk and excess fraction, although it has been given many other names [2, 3]. Such metrics are commonly reported (and misinterpreted) by the media, and often given erroneous interpretations. To clarify confusion, Greenland and Robbins distinguish PAF from ‘etiologic fractions’ that truly represent the proportion of disease prevalence that is caused by a particular risk factor [4], a quantity that can only be estimated under certain conditions Despite this misinterpretation, the attention garnered by PAF calculations signify their importance in both informing public policy regarding appropriate disease interventions and their power to influence public perception about what might and might not be healthy behaviour, or healthy levels of physiologic measures such as blood pressure.

Often, attributable fractions and their possible generalizations [5, 6] are used to rank the importance of the various risk factors that are involved in disease pathogenesis. As an example, we used attributable fractions to quantify and compare disease burden due to major stroke risk factors [7]; the analysis indicating that high blood pressure, physical inactivity and apolipoprotein levels were the most important risk factors contributing to stroke on a population level. Here, we use these same data to demonstrate an alternative and complementary graphical comparison of the importance of the risk factors under consideration. The suggested plots allow a quick visual assessment of the relative attributable fractions for differing risk factors, as well as risk factor prevalence and disease/risk factor odds ratios. The plots utilize approximations that facilitate graphical representation of PAF and impact fractions in terms of prevalence and Odds Ratio. In addition, the approximations can be used as a rule of thumb to quickly convert logistic regression coefficients into attributable fractions. Extensions of the methods to multi-category and continuous risk factors are also suggested.

## Methods

### Definition and previous estimators for PAF (binary exposures)

We first define PAF and possible estimators assuming a binary disease indicator, *Y*, and a binary risk factor (or synonymously binary disease exposure), *A*. We also state some approximations that will be used in the suggested plots, leaving their justification to the Additional file 1. While many authors have defined *PAF* using conditional probabilities for *Y* given *A*, attributable fractions are causal concepts and deserve a causal definition. With this in mind, we adopt a counterfactual notation, [8], where the pair (*Y*^*a* = 0^, *Y*^*a* = 1^) denotes the potential (or counterfactual) binary disease outcomes for an individual under the two scenarios that that they were exposed to the risk factor *A* (*a* = 1), and that they were not exposed to the risk factor *A* (*a* = 0). One interpretation of the pair (*Y*^*a* = 0^, *Y*^*a* = 1^) is that they are the disease outcomes that would be observed for that individual in two almost identical universes, which differ only according to whether that individual was exposed to the risk factor, and in the possible consequences of this exposure. In the situation that (*Y*^*a* = 0^, *Y*^*a* = 1^) = (0, 1), the risk factor, *A*, has is regarded as having a causal effect on disease for that individual . In reality, we observe either *Y*^*a* = 0^ or *Y*^*a* = 1^, but not both, as every individual (at least at a point in time) is either exposed or unexposed to *A*.

Given these preliminaries, the population attributable fraction can be defined [8] as:

E1 $$ PAF=\frac{P\left(Y=1\right)-P\left({Y}^{a=0}=1\right)}{P\left(Y=1\right)}, $$

where *P*(*Y*^*a* = 0^ = 1) can be interpreted as the disease prevalence in a population where nobody was exposed, and *P*(*Y* = 1) is current disease prevalence in the current population. While in general PAF can be negative, here we assume that the risk factor has been coded so that *P*(*Y* = 1) > *P*(*Y*^*a* = 0^ = 1), which is usually implied if *a* = 0 indicates absence of the risk factor. As explained above, *Y*^*a* = 0^ is only observed on the group of individuals who are unexposed to the risk factor, and as a result *P*(*Y*^*a* = 0^ = 1) and by extension E1 are not directly estimable. To proceed, three technical assumptions, usually referred to as consistency, positivity and conditional exchangeability, are needed (see Table 1 and [8] for further discussion). In this manuscript, we also assume no multiplicative interactions involving the exposure, or more precisely that the relative risk within a strata *c* of the confounders: *RR* = *P*(*Y* = 1| *A* = 1, *C* = *c*)/*P*(*Y* = 1| *A* = 0, *C* = *c*) does not depend on *c* [10]. Under these conditions one can rewrite E1 as follows:

E2 $$ PAF=\frac{P\left(A=1|Y=1\right)\left( RR-1\right)}{RR}. $$

**Table 1:** Definitions, assumptions and approximations for PAF when the exposure is binary, multi-category and logistic

	Binary	Multicategory	Continuous
Counterfactual definition of PAF	$\frac{P\left(Y=1\right)-P\left({Y}^{a=0}=1\right)}{P\left(Y=1\right)}$	$\frac{P\left(Y=1\right)-P\left({Y}^{a=0}=1\right)}{P\left(Y=1\right)}$	$\frac{P\left(Y=1\right)-P\left({Y}^{a={j}_0}=1\right)}{P\left(Y=1\right)}$
Assumptions:	1. Standard causal inference assumptions • Conditional exchangeability (counterfactual outcome *Y*^*a* = *j*^ and assigned risk factor *A* are independent random variables, within strata of observed confounders *c* • Consistency of counterfactuals: *Y*^*a* = *j*^ = *Y* when *A* = *j* for all levels *j* of the risk factor *A* • Positivity 0 < *P*(*Y*^*a* = *j*^ = 1| *C* = *c*) < 1 for all *j* and strata *c* 2. No interactions (*P*(*Y*^*a* = *j*^ = 1| *C* = *c*)/*P*(*Y*^*a* = *k*^ = 1| *C* = c) does not depend on *c*), for any possible values of exposure *j* and *k* 3. Rare disease assumption (P(Y = 1) small)		
Re-expression of PAF (given assumptions 1. and 2.)	*P*(*A* = 1| *Y* = 1)(*RR* − 1)/*RR*	$\sum \limits_{j=1}^KP\left(A=j|Y=1\right)\left(R{R}_j-1\right)/R{R}_j$**	${\int}_{-\infty}^{\infty }f\left(j|1\right)\frac{RR(j)-1}{RR(j)} dj$ **
^a^Corresponding logistic model (Given assumption 3.)	*logit*(*P*(*Y* = 1| *A* = *j*, *C* = *c*)) =*μ* + *β*_*j*_ + *γ*(*c*)	*logit*(*P*(*Y* = 1| *A* = *j*, *C* = *c*)) = μ + *β*_*j*_ + *γ*(*c*)	*logit*(*P*(*Y* = 1| *A* = *j*, *C* = *c*)) = μ + *β*(*j*) + *γ*(*c*)
Logistic Approximation for PAF (Given assumptions 1,2 and 3)	$\frac{\hat{P\left(A=1|Y=1\right)}\left({e}^{\hat{\beta_1}}-1\right)}{e^{\hat{\beta_1}}}$	$\sum \limits_{j=1}^K\hat{P}\left(A=j|Y=1\right)\left({e}^{\hat{\beta_j}}-1\right)/{e}^{\hat{\beta_j}}$	${\int}_{-\infty}^{\infty}\hat{f}\left(j|1\right)\left({e}^{\hat{\beta (j)}}-1\right)/{e}^{\hat{\beta (j)}} dj$***
Graphical Approximation	$\hat{P\left(A=1|Y=0\right)}\times {\hat{\ \beta}}^{ave}$	$\hat{P}\left(A>0|Y=0\right)\times {\hat{\ \beta}}^{ave}$	$1\times {\hat{\beta}}^{ave}$****
“Average” estimated log-odds ratio: ${\hat{\beta}}^{ave}$	$\hat{\beta_1}$	$\frac{\sum \limits_{j=1}^K\hat{P}\left(A=j|Y=0\right)\hat{\beta_j}}{1-\hat{P}\left(A=0|Y=0\right)}$	${\int}_{-\infty}^{\infty}\hat{f}\left(j|0\right)\hat{\beta (j)} dj$

*Here *β*_0_ = 0 by definition for the Binary and Multicategory exposures and *β*(*j*_0_) = 0 for continuous exposures. Estimates $\hat{\beta}(j)/{\hat{\beta}}_j\ \mathsf{and}$ $\hat{\gamma}(c)$ could be found via generalized additive models with a logistic link, where the confounders and possibly the exposure are modelled non-parametrically

**Note that *RR*_*j*_ = *P*(*Y* = 1| *A* = *j*, *C* = *c*)/*P*(*Y* = 1| *A* = 0, *C* = *c*) and *RR*(*j*) = *P*(*Y* = 1| *A* = *j*, *C* = *c*)/*P*(*Y* = 1| *A* = *j*_0_, *C* = *c*)

***f(*j*| 1) is the conditional density of *A* when Y = 1; similarly *f*(*j*| 0) is the conditional density of *A* when Y = 0

****Note that when *A* is continuous, the probability of a non-reference level of the exposure: $\hat{P}\left(A\ne {j}_0|Y=0\right)$ is 1

Note that under the same conditions other estimable expressions for E1 do exist (see (3)), but E2, an expression that was first derived in [9], has the added attraction of estimability in case-control studies. A short proof of the equality of E1 and E2 under these assumptions is provided for convenience in the Additional file 1, but similar results have been proven already elsewhere [17, 18].

Under an additional assumption that the disease risk is small under each strata *c* of the confounders, the conditional odds ratio: *OR* = *Odds*(*Y* = 1| *A* = 1, *C* = *c*)/*Odds*(*Y* = 1| *A* = 0, *C* = *c*) where *Odds*(*Y* = 1| *A* = a, *C* = *c*) = *P*(*Y* = 1| *A* = *a*, *C* = *c*)/(1 − *P*(*Y* = 1| *A* = 0, *C* = *c*)) is a close approximation for *RR.* This implies that under this ‘rare disease’ assumption, PAF can be then estimated by substituting an estimated Odds Ratio, $\hat{OR}$, that is adjusted for *c,* and the sample proportion of cases with *A* = 1, $\hat{P}\left(A=1|Y=1\right)$, into E2 . Typically, $\hat{OR}$ is then calculated via exponentiating the coefficient for the risk factor, $\hat{\beta_1}$, in a logistic regression model (see Table 1) that regresses *Y* against *A* and *C* leading to the estimator:

E2b $$ \hat{PAF}=\frac{\hat{P}\left(A=1|Y=1\right)\ \left({e}^{\hat{\beta_1}}-1\right)}{e^{\hat{\beta_1}}}. $$

This approach described above has formed the backbone of many previous attributable fraction estimators [11, 12]. In the Additional file 1, we derive the following approximation for E. 2b:

E2c $$ \hat{PAF}\sim \hat{P}\left(A=1|Y=0\right)\ \hat{\beta_1} $$

implying that the estimated PAF is approximately the estimated log-odds ratio between the risk factor and disease multiplied by the estimated prevalence of the risk factor in controls.

### Definition of PAF for multicategory and continuous exposures

These definitions and results extend easily to multicategory and continuous exposures. For instance, suppose that the exposure *A* can take *K* + 1 values: *a* ∈ 0, 1, …, *K*, with *a* = 0 a reference level such that:

E3 $$ P\left({Y}^{a=j}=1\right)\ge P\left({Y}^{a=0}=1\right) $$

for aIl *j* = 1, …, *K*. In this case, the formula for PAF is still given by E1 which now has the interpretation as the proportion of disease cases removed in a hypothetical population where everyone had *A* = 0. In the case that *A* is continuous, we set the A = *j*_0_ to be a minimum risk level of the exposure variable, that is:

E4 $$ P\left({Y}^{a=j}=1\right)\ge P\left({Y}^{a={j}_0}=1\right) $$

for all possible exposure values: *j*. Here, a suitable definition of PAF is the following:

E5 $$ PAF=\frac{P\left(Y=1\right)-P\left({Y}^{a={j}_0}=1\right)}{P\left(Y=1\right)}, $$

and has the interpretation as the proportion of disease cases removed in a hypothetical population where everyone had *A* = *j*_0_. Note that in order to estimate E5, *j*_0_ needs to be a realizable value of the exposure variable, with sufficient data in its vicinity to estimate relative risks. For instance, *j*_0_ = 0 would not be an acceptable value of systolic blood pressure, even if the relationship between blood pressure and disease risk was strictly increasing.

For both multicategory and continuous exposures, the appropriate estimators for PAF, underlying assumptions and possible approximations are similar to those described in the binary case above and are detailed in Table 1, and proven in the Additional file 1. In particular, we still have a result with a similar flavour to (2c):

E6 $$ \hat{PAF}\sim \hat{\mathrm{P}}{\hat{\beta}}^{ave}, $$

with $\hat{P}$ now the estimated probability of an individual having a non-reference level of the exposure in controls and ${\hat{\beta}}^{ave}$ an average of estimated log-odds ratios for various exposure levels of *A* compared to the reference weighted according to the distribution of exposure in controls. Provided ${\hat{\beta}}^{ave}$ is not too large and the disease is rare, another re-interpretation of ${\hat{\beta}}^{ave}$ is it is approximately the average percentage elevation in risk when comparing the actual exposure levels observed in the population to the reference exposure. Note that *E*6 reduces to *E*2*c* in the case that the risk factors are binary.

## Results

### Application of approximations on INTERSTROKE

INTERSTROKE [7] is a large international case control study designed to quantify the contribution of established risk factors to stroke prevalence at a global level. Here we consider the 10 major risk factors for stroke considered in [7]. These are high blood pressure, waist-to-hip ratio, diet, physical activity, diabetes mellitus, alcohol intake, stress, presence of cardiac risk factors and the ratio of apolipoproteins B to A1. In the original analysis, waist hip ratio, diet score, alcohol intake and Apo-A/Apo-B ratio were divided into 3 groups, necessitating a multi-category approach to calculating attributable fractions for these variables. To apply the approximations described above, we first coded the minimum risk group for each risk factor, which was known for the 10 risk factors we consider here, as *a* = 0. Conditional Odds Ratios were then estimated via a logistic regression model that adjusted for age, sex and country as well as the other 9 risk factors. Note that stroke, while regarded as a common disease, only has a prevalence of around 1% in the population [13] indicating that the PAF approximations derived above may be appropriate. Table 2 shows the log-Odds Ratios, Odds Ratios, prevalences (in controls) and the approximation E6 together with the exact calculation given by the logistic approximation in Table 1. The approximation is acceptably accurate, except perhaps for cardiac causes, the risk-factor having the largest estimated odds ratio. Later, we will describe more precisely when the approximations may break down.

**Table 2:** Illustration of the approximations on the INTERSTROKE dataset. For binary risk factors, ${\hat{\beta}}^{ave}=\mathit{\log}\left(\hat{OR}\right)$, for multicategory risk factors ${\hat{\beta}}^{ave}$ is a kind of weighted average log odds ratio summarizing the increase in risk of non-reference levels of the risk factor compared to the reference level. Confidence intervals for exact PAF are given at 99% level and calculated using Bootstrap

Risk factor	${\hat{\beta}}^{ave}\sim \mathit{\log}\left(\hat{OR}\right)$	${e}^{{\hat{\beta}}^{ave}}\sim \hat{OR}$	prevalence exposure in controls	Approximate PAF: [7]	Exact calculation PAF: [6]
High blood pressure (Y/N)	1.093	2.98	47.4%	51.8%	47.9% (45.1–50.6)
Lack of physical activity	0.501	1.65	83.7%	41.9%	35.5% (27.7–44.7)
ApoA, ApoB ratio (in tertiles)	0.428	1.53	66.9%	28.6%	26.9% (22.2–31.9)
Diet score (in tertiles)	0.378	1.46	67.0%	25.3%	23.0% (18.2–28.9)
Waist hip ratio (in tertiles)	0.294	1.34	67.0%	19.7%	18.8% (13.3–25.3)
Smoking (Y/N)	0.513	1.67	22.4%	11.5%	12.4% (10.2–14.9)
Cardiac causes (Y/N)	1.156	3.18	4.9%	5.7%	9.1% (8.0–10.2)
Frequency of alcohol consumption (3 levels)	0.186	1.20	27.7%	5.2%	5.9% (3.4–9.7)
Global stress (Y/N)	0.301	1.35	14.4%	4.3%	5.0% (2.6–7.3)
Diabetes (Y/N)	0.148	1.16	12.9%	1.9%	2.4% (0.1–4.9)

### Simultaneous graphical representation of PAF, odds ratios and prevalence

While it is well recognized that PAF depends jointly on risk factor prevalence and relative risk, the relative contributions of these two components may be hidden when PAF is reported. A typical solution is to psresent PAF, risk factor prevalence and estimated odds ratios in some summary table [7]. Nevertheless, for some readers a single graph, simultaneously representing all three metrics might convey the same information in a more efficient, intuitive and visually appealing manner. For instance, clusters of risk factors with similar log-odds ratio and prevalence are more easily identifiable using these graphs. Here we use the approximations described above to construct such plots. Denoting the approximate PAF from E6 as ${\hat{PAF}}_a=\hat{P}{\hat{\beta}}^{ave}$, the first plot involves the re-expression:

E7 $$ {\hat{\beta}}^{ave}=\frac{\hat{PA{F}_a}}{\hat{P}}. $$

Imagine now a set of *N* disease risk factors (either binary, multi-category or continuous); we denote the inverse prevalence, log-OR pair for the i^th^ risk factor as $\left({\hat{P}}_i^{-1},{\hat{\beta}}_i^{ave}\right)$ . Plotting the $\left({\hat{P}}_i^{-1},{\hat{\beta}}_i^{ave}\right)$ pairs on a standard x-y co-ordinate axis, risk factors with inverse prevalence/log-odds ratio pairs lying on the line of slope *K*: ${\hat{\beta}}^{ave}=K/\hat{P}$ emanating from the origin both have the same approximate attributable fractions, $\hat{PA{F}_a}=K$. Note that binary, multicategory and continuous exposure variables can all be represented on this same axis, with the understanding that ${\hat{P}}_i$ represents prevalence of the risk factor (in the binary case), and the prevalence of a ‘risk-increasing’ level of the exposure (in the multicategory and continuous cases). The resulting plot resembles a fan, with risk factors bearing heavier disease burden lie on lines of increasing slope. The slope of any such line is an approximate attributable fraction. Another observation regarding equation *E7:* $\mathrm{y}=\hat{PA{F}_a}.\frac{1}{\hat{\left(\mathrm{P}\right)}}$, is that $\mathrm{y}=\hat{PA{F}_a}$ when $\hat{P}=1$, implying that if we move the y-axis to $1/\hat{\mathrm{P}}=1$, the y-intercept of the line emanating from (0,0) to $\left({\hat{P}}_i^{-1},{\hat{\beta}}_i^{ave}\right)$ will be the approximate PAF.

**Fig. 1:**
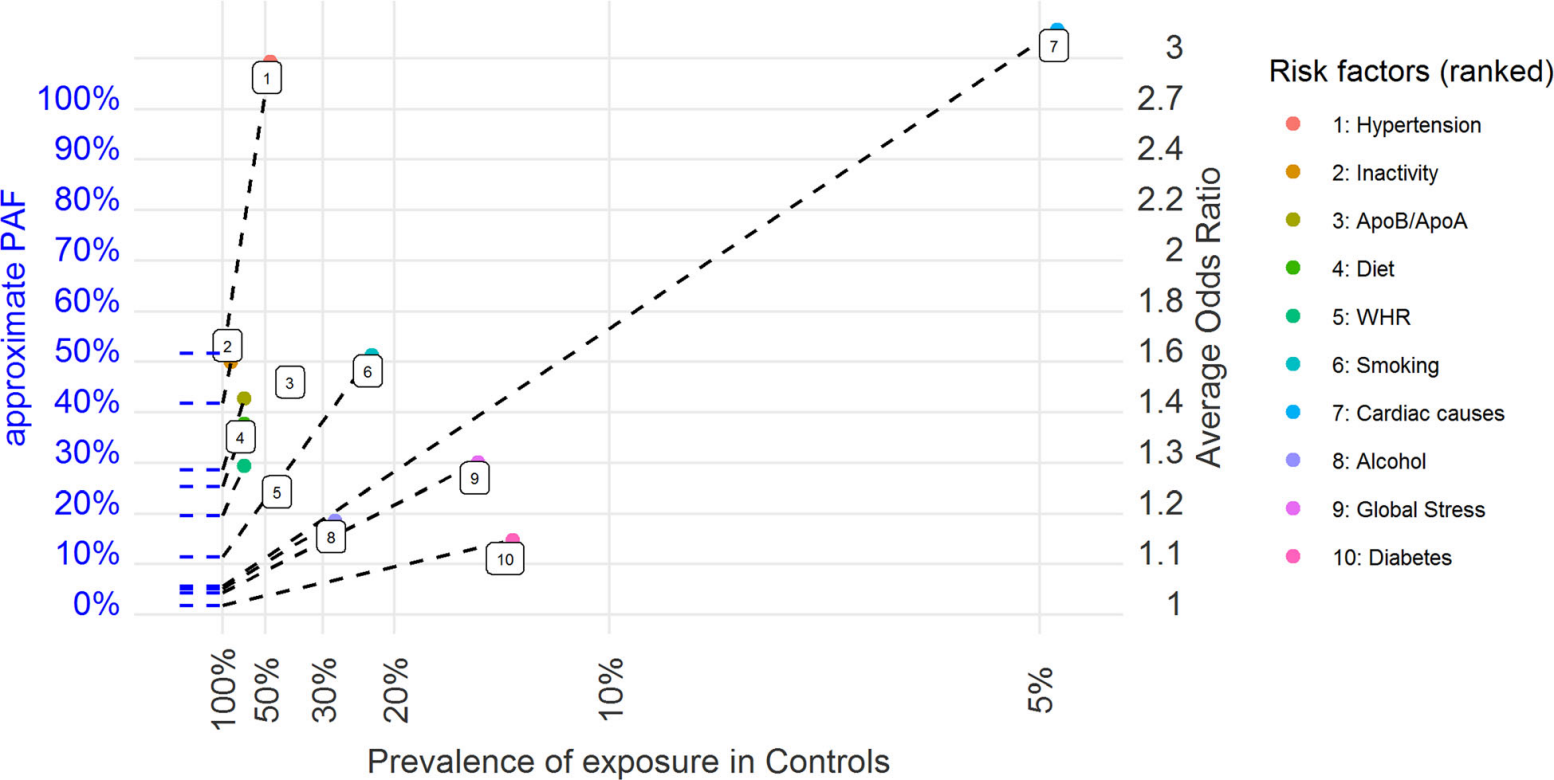
Graphical representation of estimates of approximate PAF (left-hand y-axis), prevalence (x-axis) and odds ratios (right-hand y-axis) for 10 risk factors from the INTERSTROKE dataset. Approximate PAF is represented both by the slope of the black dashed line, and also the left y-axis intercept of the same line. Prevalence and Odds Ratio information is displayed as on a usual scatterplot (although prevalence decreases from left to right). Risk factors are ranked 1–10 according to approximate PAF

Figure 1 demonstrates a figure built on these observations for all 10 rows of Table 2. The plot allows simultaneous visual representation and ranking of the prevalence, odds ratio and attributable fraction for a number of risk factors. For example, the largest attributable fraction (hypertension) is both represented as the line of greatest slope, and as the largest y-intercept. The other 2 axes represent prevalence (x-axis) and odds ratios (y-axis on right hand side). Note that Cardiac causes (such as prior atrial fibrillation or myocardial infarction) stand on the plot as having the highest odds ratio of stroke but contributes only modestly to stroke burden due to the low prevalence of these causes in the population.

### Attributable fraction nomograms

**Fig. 2:**
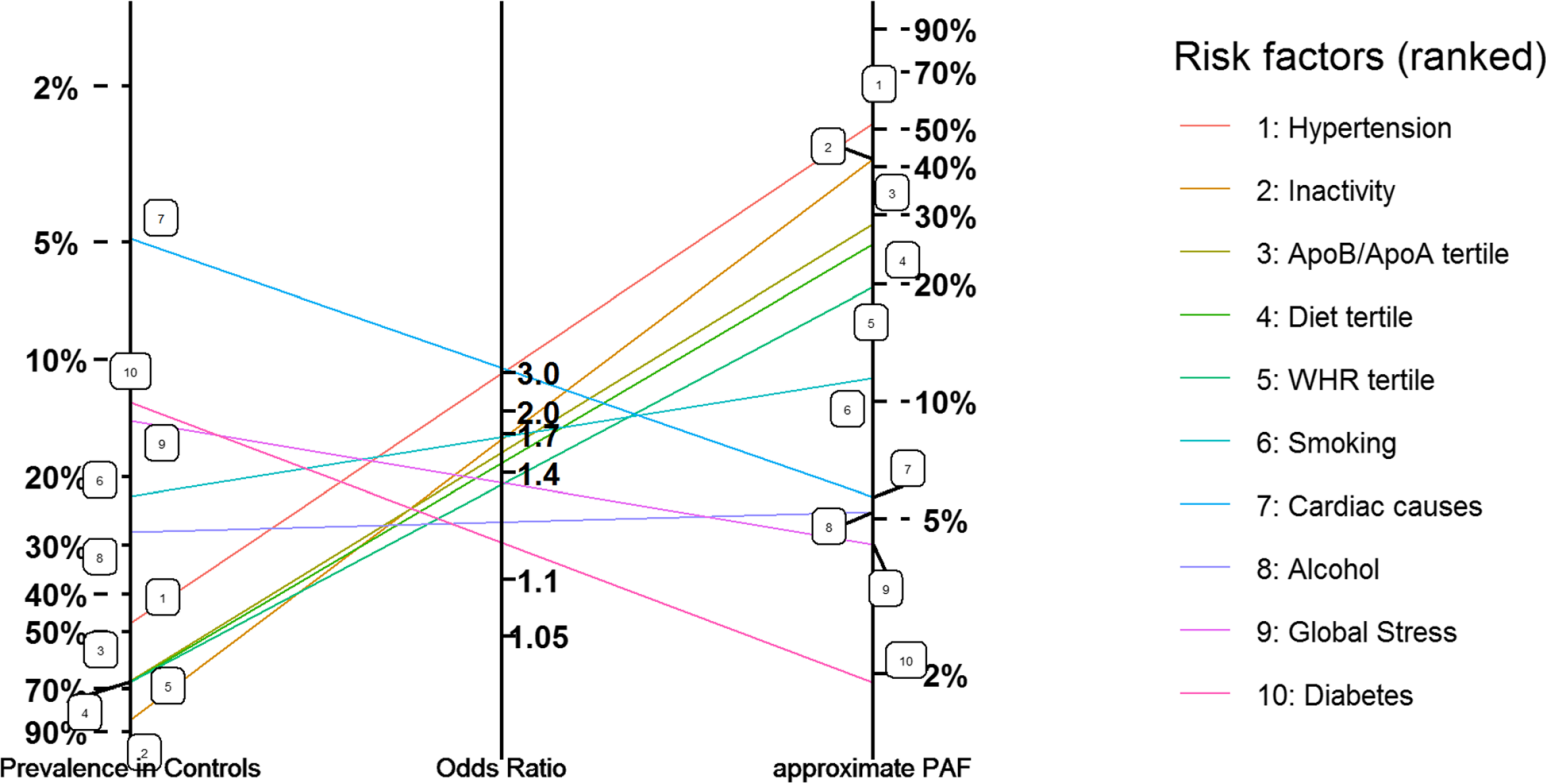
Attributable fraction nomogram displaying estimates of risk factor prevalence, average Odds Ratio and approximate PAF for the 10 INTERSTROKE risk factors. The prevalence, OR and approximate PAF for a particular risk factor are identified on the same line. Again risk factors are ranked according to approximate PAF, recorded on the right-most axis

Interestingly, the multiplicative formula (E6) to produce an approximate attributable fraction from prevalence in controls and an estimate of the log odds ratio/relative risk is similar to the updating rule in Bayes Rule (that is, prior odds x Likelihood = posterior odds), a dependence that has been exploited in the nomograms suggested by Fagan [14]. This analogy suggests a similar pictorial representation can be used to display the relationships between approximate PAF, prevalence and the estimated log-OR (${\hat{\upbeta}}^{ave}$). In more detail, the multiplicative equation (E6) transforms to a linear equation upon taking logs of both sides of the equation, with the result that $\log \Big({\hat{\upbeta}}^{ave}$) is proportional to the average of the log prevalence (in controls) and the log approximate PAF. This observation facilitates a log-scale plot where control prevalence, odds ratios and approximate PAFs for each risk factor are connected with lines (see Fig. 2). Here clustering of risk factors (having similar prevalences, odds ratios and approximate PAFs) are represented as effective equality of the corresponding lines (for instance WHR tertile and diet tertile in Fig. 2). When 2 risk factors have similar PAF, but have differing prevalences and relative risks, the corresponding lines have very different slopes but will almost intersect at the same approximate PAF vertical (an example being cardiac-related risk factors and alcohol in Fig. 2).

**Fig. 3:**
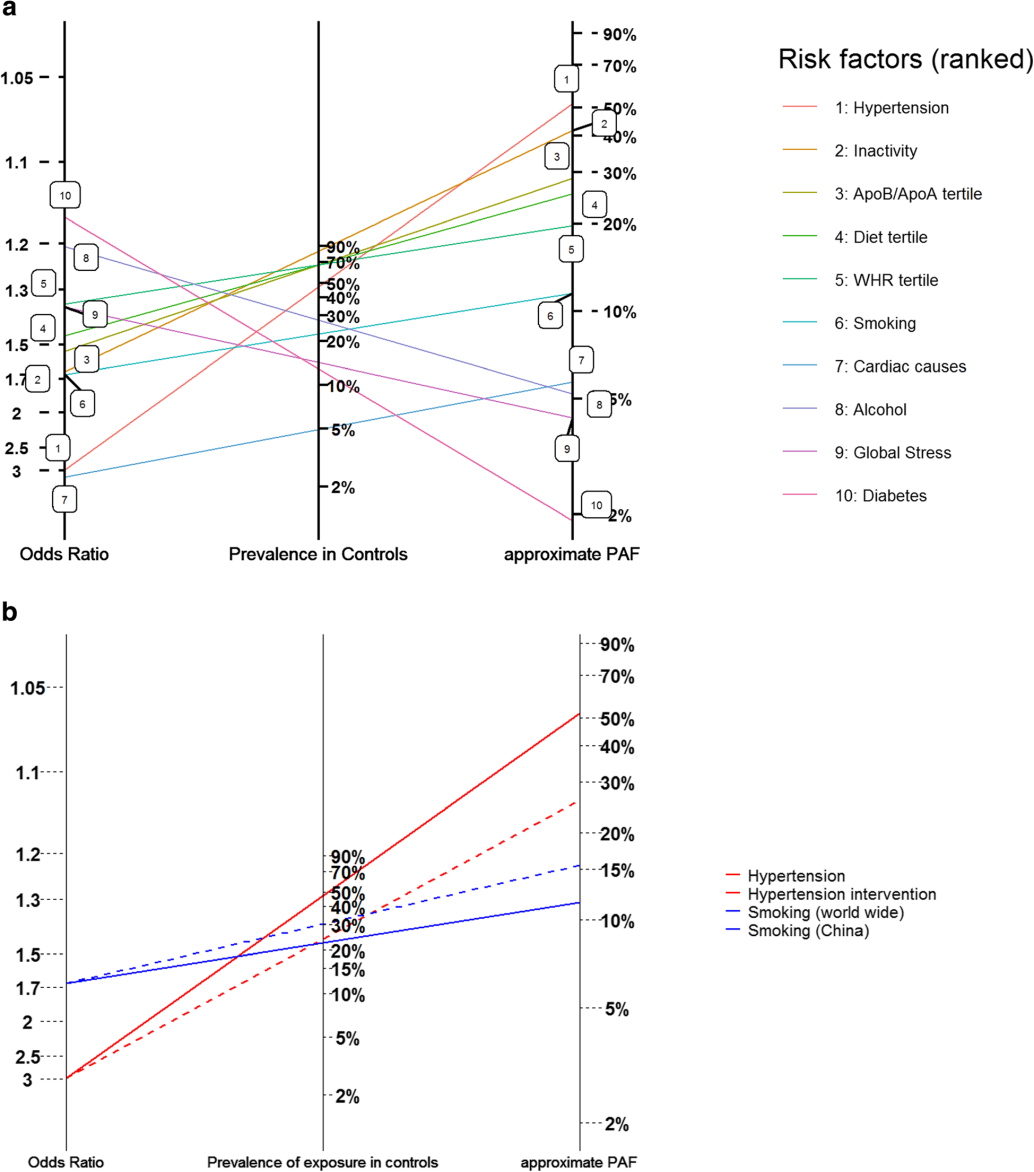
**a/b** Alternative formatting of attributable fraction nomogram displaying estimates of risk factor prevalence, average Odds Ratio and approximate PAF for the 10 INTERSTROKE risk factors. The prevalence, OR and approximate PAF for a particular risk factor are identified on the same line. Here the left hand axis records estimated average odds ratios and the middle axis records estimated prevalence. Differing interventions that might reduce risk factor prevalence might be compared by rotating the line for a given risk factor using the left axis intercept as a pivot. For example, in the bottom pane, the difference in approximate PAF between the red dashed line and the solid line estimates the % reduction in the prevalence of stroke from an intervention that halved the prevalence of hypertension. The blue dashed line estimates the PAF for smoking in China (where the prevalence of smoking is higher than the global average)

The symmetry of the multiplicative relationship (E6) allows us to reorder the prevalence and odds ratios axes of the previous nomogram, so the left axis represents Odds Ratio, with the middle axis now representing control prevalence (Fig. 3a). This alternative representation may be helpful as the lines originating at a particular OR can be ‘tilted’ to represent alternative populations (where the prevalence of the risk factor might be higher or lower), or perhaps instead a partial elimination of the prevalence of the risk factor resulting from an intervention (that is an impact fraction, rather than PAF). For example, Fig. 3b shows the approximate PAF that would result if the prevalence of smoking was 30.2% (as calculated in Chinese INTERSTROKE controls) compared to 22.4%, and the approximate impact fraction that would result from an intervention that halved the prevalence of hypertension (effectively this is the approximate PAF for hypertension if the prevalence was 0.474/2 = 0.237 rather than 0.474).

### Biases in approximations for larger odds ratios

**Fig. 4:**
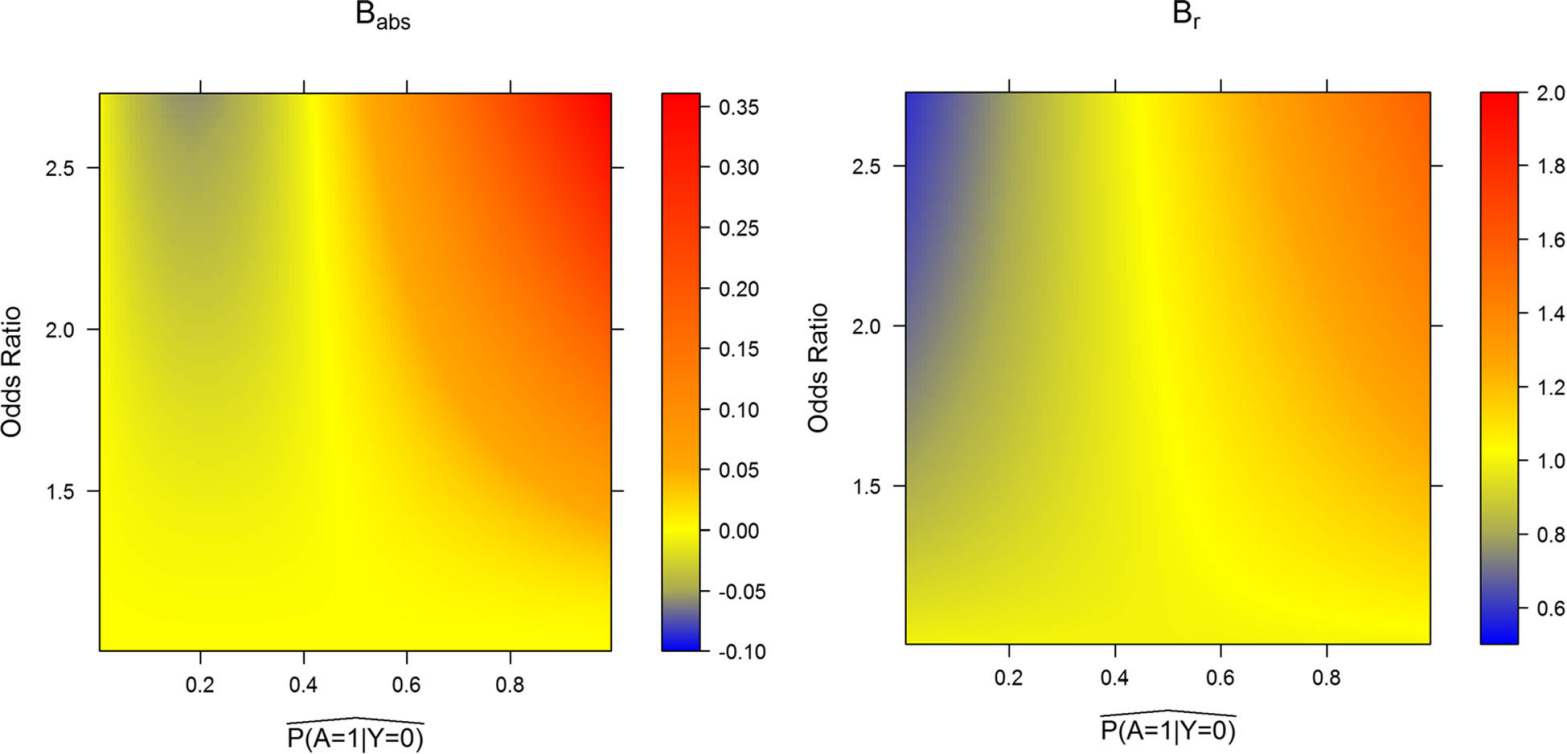
Absolute and relative bias from approximations as functions of the estimated prevalence and estimated odds ratios of the risk factor. Babs is defined as PAFa -PAF, with PAF being the usual estimate of PAF and PAFa the estimated approximate PAF defined in this manuscript. Br is defined as PAFa/PAF

The approximations described here are derived from second order Taylor expansions of (2b) and its analogues for multi-category and continuous exposures. These are derived and analysed in detail in the Additional file 1. Figure 4 describes the estimated biases, both as a ratio: ${\mathrm{B}}_{\mathrm{r}}=\frac{\hat{\mathrm{PA}{\mathrm{F}}_{\mathrm{a}}}}{\hat{\mathrm{PA}\mathrm{F}}}$ and as an absolute value: ${\mathrm{B}}_{\mathrm{a}\mathrm{bs}}=\hat{\mathrm{PA}{\mathrm{F}}_{\mathrm{a}}}-\hat{\mathrm{PA}\mathrm{F}}$, where the exact and approximate values are calculated using E2b and E2c. The Taylor expansion effectively creates a quadratic function of $\hat{\beta_1}$ that closely approximates E2b for small $\hat{\beta_1}$. If $\hat{\beta_1}<0.4$, corresponding to an odds ratio of 1.5, the approximation will be reasonably accurate (within 20% on an exact calculation), regardless of the prevalence of the risk factor. For odds ratios smaller than 3, these approximations can still be used, if the prevalence of the risk factor is less than 60% (particularly if absolute bias in PAF quantification is more important than relative bias quantification). For larger odds ratios, the approximation may be inaccurate. Interestingly, the approximation tends to be most accurate when the prevalence is controls is approximately 0.5. Arguments as to why this should be the case are presented in the Additional file 1.

## Discussion

The graphical approaches described in this manuscript facilitate the visual assessment of relative risk factor burden according to a number of different criteria on a single axis. The plots depend on a simple approximation formula for PAF, that may be of interest in of itself, both as a quick rule of thumb to calculate PAFs and impact fractions, and in that it demonstrates that risk factor prevalence and risk factor/disease log-odds ratio equally contribute to PAF. The 2 plots proposed both have their advantages and disadvantages. While both methods allow detection of risk factor clusters having similar prevalence and odds ratios, it is more natural to visualize clustering on a natural 2-dimensional x-y plane as in Fig. 1, than it is on a nomogram in Fig. 2. Conversely, while both methods offer an explanation as to why a certain risk factor has a particular PAF, perhaps the representation given by the nomogram lends extra intuition to some epidemiologists who are already familiar with the use of likelihood ratio nomograms in diagnostic testing. Admittedly, these plots have limitations. The inverse or log-scaling used may create confusion regarding the absolute differences in PAF between the different risk factors. For instance, there is a larger difference in the PAFs for hypertension and physical inactivity than Fig. 1 might suggest since the log-scaling has distorted the absolute difference in PAF. Second, the approximations derived are only valid for logistic disease models, with no effect modification between the risk factors and confounders. A third problem is that the approximations used may be inaccurate for larger odds ratios. These limitations indicate that the plots might be best used as a visual accompaniment to, and not a replacement for, exact calculations of attributable fractions. A final point is that the suggested graphs can be used to compare continuous and discrete risk factors on the same axis. Often naturally continuous risk factors such as blood pressure are discretized for clinical convenience and interpretability; but whether it is fair to rank the PAFs for artificially discretized risk factors against un-discretised continuous risk factors is questionable. For instance, categorizing a naturally continuous risk factor into two groups only makes statistical sense if there is a threshold effect, where the risk suddenly ‘jumps’ at the threshold separating the categories. Otherwise discretization can be a very crude approximation and is likely to disadvantage a risk factor in a ranking compared with continuous risk factors that have not be categorized.

The approximations derived will work well in genetic settings, where Odds Ratios tend to be low. Even though genetic variables (such as single nucleotide polymorphisms) are not modifiable, attributable fractions are still of interest and have been used as a measure of disease heritability in some settings [6, 15]. In contrast, while the Odds Ratios in INTERSTROKE are larger, the approximate calculations are perhaps acceptably accurate (Table 1). However, extremely large odds ratios are possible in traditional epidemiologic applications. For instance, the odds ratio linking smoking and lung cancer was initially estimated to be roughly 9 [16]. While in these extreme cases the approximate PAF will be unacceptable as a proxy for an exact calculation (and may indeed be larger than 1), the plots suggested here may still convey a robust measure of risk factor importance, provided the absolute quantification of PAF is not of interest.

## Conclusions

The described methods could be used as a complement to tables summarizing prevalence, odds ratios and PAF, and may convey the same information in a more intuitive and visually appealing manner. The suggested nomogram can also be used to visually estimate the effects of health interventions which only partially reduce risk factor prevalence. Finally, in the binary risk factor case, the approximations can also be used to quickly convert logistic regression coefficients for a risk factor into approximate PAFs.

## Additional file

Additional file 1: Graphical comparisons of relative disease burden across multiple risk factors. (PDF 144 kb)

## Data Availability

The data to reproduce the examples in the manuscript is given in Table 2. R-code to reproduce the plots can be obtained by contacting the corresponding author directly.

## Abbreviations

OR: Odds Ratio
PAF: Population attributable fraction
RR: Relative risk
