# Supplementary material for: Graphical comparisons of relative disease burden across multiple risk factors
Source: BMC Med Res Methodol. 2019 Sep 11;19:186. doi: 10.1186/s12874-019-0827-4 (PMC6737608; doi:10.1186/s12874-019-0827-4)
Supplement: Supplementary file 1 — Graphical comparisons of relative disease burden across multiple risk factors. (PDF 144 kb) [file 12874_2019_827_MOESM1_ESM.pdf]

Supplementary information

Graphical comparisons of relative disease burden across multiple risk factors

1 Technical details on the approximations

Again, we first consider the approximations for a binary risk factor, A, and later generalize. Consider a second order Taylor expansion of: $\left(e^{\widehat{\beta_1}}-1\right)/e^{\widehat{\beta_1}}=1-e^{-\widehat{\beta_1}}$ around $\widehat{\beta_1}$ = 0. That is: $1-e^{-\widehat{\beta_1}} \sim 1-\left(1-\widehat{\beta_1}+\widehat{\beta_1^2}/2\right)$ = $\widehat{\beta_1}\left(1-\widehat{\beta_1}/2\right)$. Plugging this approximation into (2b) yields:

$$\widehat{\mathrm{PAF}} \sim \widehat{\mathrm{P}(\mathrm{A}=1|\mathrm{Y}=1)}\widehat{\beta_1}\left(1-\widehat{\beta_1}/2\right). \tag{S1}$$

(3) is still slightly unwieldly for our purposes; a couple of further steps yields simpler expressions:

(a) For small $\widehat{\beta_1}$, ignoring, we can ignore the second order factor (S1) to derive:

$$\widehat{\mathrm{PAF}} \sim \widehat{\mathrm{P}(\mathrm{A}=1|\mathrm{Y}=1)}\widehat{\beta_1}. \tag{S1a}$$

In practice, this first-order approximation may be inacceptably inaccurate, and is not recommended for use. For instance, to achieve an acceptable approximation (perhaps defined as the ratio of (S1) and (S1a)) being within 20%), we can solve $\left(1-\widehat{\beta_1}/2\right) > 0.8$, leading to $\widehat{\beta_1} < 0.4$, implying that this approximation should be used for risk factors with odds ratios less than 1.5.

(b) If $|\widehat{\beta_1}|$ is larger, we may need to keep the quadratic factor in (S1) to obtain an acceptable approximation. To derive an alternative approximation, note that (S1) can be rewritten as $\widehat{\mathrm{PAF}} \sim \widehat{\beta_1}\mathrm{F}$ wher $\mathrm{F}=\widehat{\mathrm{P}(\mathrm{A}=1|\mathrm{Y}=1)}\left(1-\widehat{\beta_1}/2\right)$

It follows that:

$$\mathrm{F}=\widehat{\mathrm{P}(\mathrm{A}=1|\mathrm{Y}=0)}\frac{\widehat{\mathrm{P}(\mathrm{A}=1|\mathrm{Y}=1)}}{\widehat{\mathrm{P}(\mathrm{A}=1|\mathrm{Y}=0)}}\left(1-\widehat{\beta_1}/2\right)$$

$$=\widehat{\mathrm{P}(\mathrm{A}=1|\mathrm{Y}=0)}\frac{\widehat{\mathrm{P}(\mathrm{Y}=1|\mathrm{A}=1)}/\mathrm{P}\widehat{(\mathrm{Y}=1)}}{\widehat{\mathrm{P}(\mathrm{Y}=0|\mathrm{A}=1)}/\mathrm{P}\widehat{(\mathrm{Y}=0)}}\left(1-\widehat{\beta_1}/2\right)$$

$$\sim\widehat{\mathrm{P}(\mathrm{A}=1|\mathrm{Y}=0)}\frac{\widehat{\mathrm{P}(\mathrm{Y}=1|\mathrm{A}=1)}}{\mathrm{P}\widehat{(\mathrm{Y}=1)}}\left(1-\widehat{\beta_1}/2\right),$$

with the equality in line 2 following by Bayes theorem and the final approximation by the rare disease assumption.

Now if $\widehat{\beta_1} > 0$, we would expect that $\widehat{P(Y=1|A=1)}/\widehat{P(Y=1)} > 1$ (provided confounding isn't reversing the conditional and unconditional directions of association), and for the same reason when $\widehat{\beta_1} < 0$ it is likely that $\widehat{P(Y=1|A=1)}/\widehat{P(Y=1)} < 1$. Under the scenario that the 2nd and 3rd terms in (3) are approximate reciprocals:

$$\frac{\widehat{P(Y=1|A=1)}}{\widehat{P(Y=1)}}\left(1-\widehat{\beta_1}/2\right)\sim 1 \qquad \text{(S1b)}$$

and the two-term expansion given in (3) can be approximated by (3c):

$$\widehat{PAF}\sim \widehat{P(A=1|Y=0)}\widehat{\beta_1}. \qquad \text{(S1c)}$$

That is, an approximation for the population attributable fraction is the prevalence of the exposure in controls multiplied by the log-odds ratio of the association.
As (S1c) may approximate the 2nd order Taylor expansion, it will usually be more accurate than (S1a), which is the first order approximation. See section 2 for a justification of when this might be most accurate.

Approximations for multicategory and continuous exposures.

The previous approximations extend easily to multicategory exposures. Suppose that the exposure A can take $K+1$ values: $a \in 0,1,\dots,K$ with $a=0$, a reference level such that:

$$P\left(Y^{a=j}=1\right) \geq P(Y^{a=0}=1) \qquad \text{(S2)}$$

for all j. Under the assumptions that (a) adjusting for the set of variables, $\mathbf{C}$, is sufficient to eliminate confounding of the exposure/outcome relationship, that is $Y^{a=j}$, that is $A \perp Y^{a=j}|C$ for all j (b) that the relative risk $P\left(Y^{a=j}=1|C=c\right)/P(Y^{a=0}=1|C=c) = P(Y=1|A=j,C=c)/P(Y=1|A=0,C=c) = RR_j$ is independent of c for all j, (1) can be re-expressed as:

$$PAF = \sum_{j=1}^{K} P(A=j|Y=1)\left(RR_j-1\right)/RR_j\,. \qquad \text{(S3)}$$

Under the rare disease assumption, the absence of an interaction (between A and C) suggests that the conditional association between Y and A given c should be modelled via logistic regression:

$$\text{logit}\left(P(Y=1|\,A=j,C=c)\right) = \beta_0+\beta_j+f(c),$$

for j ≥ 1. (9) can then be estimated as:

$$\widehat{\mathrm{PAF}} = \sum_{j=1}^{K} \mathrm{P}(\mathrm{A} = j|\mathrm{Y} = 1)\left(e^{\widehat{\beta_j}} - 1\right)/e^{\widehat{\beta_j}}. \quad \text{(S4)}$$

Assuming 'small' β_j, $j \leq K$, each term in (S4) can in turn be approximated via Taylor expansion as before (when deriving (S1c)):

$$\widehat{\mathrm{PAF}} \sim \sum_{j=1}^{K} \widehat{\mathrm{P}(\mathrm{A} = j|\mathrm{Y} = 0)}\widehat{\beta_j} \quad \text{(S5)}$$

Finally, consider a continuous exposure, A, taking any possible value on the real line. Suppose that j_0 is a minimum risk level of the exposure such that:

$$\mathrm{P}\left(\mathrm{Y}^{a=j} = 1\right) \geq \mathrm{P}\left(\mathrm{Y}^{a=j_0} = 1\right) \quad \text{(S6)}$$

In this case, assuming again no interaction between the exposure, A and C on the relative risk scale, and that adjustment for C is sufficient to eliminate confounding, we can re-express (E5) from the main manuscript as

$$\mathrm{PAF} = \int_{-\infty}^{\infty} f(j|1)\frac{\mathrm{RR}(j) - 1}{\mathrm{RR}(j)}\,dj, \quad \text{(S7)}$$

where $\mathrm{P}\left(\mathrm{Y}^{a=j} = 1|\mathrm{C} = c\right)/\mathrm{P}\left(\mathrm{Y}^{a=j_0} = 1|\mathrm{C} = c\right) = \mathrm{P}(\mathrm{Y} = 1|\mathrm{A} = j, \mathrm{C} = c)/\mathrm{P}(\mathrm{Y} = 1|\mathrm{A} = j_0, \mathrm{C} = c) = \mathrm{RR}(j)$. We assume again an additive model relating the response to exposure and possible confounders,C:

$$\mathrm{logit}\left(\mathrm{P}(\mathrm{Y} = 1|\ \mathrm{A} = j, \mathrm{C} = c)\right) = \beta_{j_0} + \beta(j) + f(c), \quad \text{(S8)}$$

where $\beta(j)$ is a continuous positive function on the real line satisfying $\beta(j_0) = 0$.

The same approximations work again, except that the sums in (S5) now turns into an integral:

$$\widehat{\mathrm{PAF}} \sim \int_{-\infty}^{\infty} \widehat{f(j|0)}\widehat{\beta(j)}\,dj, \quad \text{(S9)}$$

where $\widehat{f(j|0)}$ is a estimated density functions (with respect to Lebesgue measure) for A in controls (i.e. when $Y = 0$).

2 When is the approximation most accurate?

The approximation (S1b):

$$\frac{\widehat{P(Y=1|A=1)}}{\widehat{P(Y=1)}}\left(1-\widehat{\beta_1}/2\right)\sim 1 \qquad \text{(S1b)}$$

was used to justify (S1c), which is the approximation used in the plots. This suggests that the error of approximating the second order Taylor expansion $\widehat{P(A=1|Y=1)}\widehat{\beta_1}\left(1-\widehat{\beta_1}/2\right)$ with $\widehat{PAF}\sim\widehat{P(A=1|Y=0)}\widehat{\beta_1}$ should be minimal when

$$\frac{\widehat{P(Y=1|A=1)}}{\widehat{P(Y=1)}}=\left(1-\widehat{\beta_1}/2\right)^{-1} \qquad \text{(S10)}$$

When is (S10) approximately true? Well, the left hand side (LHS) can be re-expressed as:

$$\text{LHS}=\left(\frac{\widehat{P(A=1)}\widehat{P(Y=1|A=1)}+\widehat{P(A=0)}\widehat{P(Y=1|A=0)}}{\widehat{P(Y=1|A=1)}}\right)^{-1}$$

$$=\left(\widehat{P(A=1)}+\widehat{RR}^{-1}\widehat{P(A=0)}\right)^{-1}=\frac{\widehat{RR}}{\widehat{RR}\widehat{P(A=1)}+\widehat{P(A=0)}}$$

$$\sim\frac{e^{\hat\beta}}{e^{\hat\beta}\widehat{P(A=1)}+\widehat{P(A=0)}}$$

$$\sim\frac{1+\hat\beta}{(1+\hat\beta)\widehat{P(A=1)}+\widehat{P(A=0)}}$$

the last approximation via a first order Taylor approximation of $e^{\hat\beta}$ around $\hat\beta=0$

Where as the RHS is:

$$\text{RHS} = \left(1-\widehat{\beta_1}/2\right)^{-1}\sim 1+\beta/2$$

again via a first order Taylor approximation of $\left(1-\widehat{\beta_1}/2\right)^{-1}$around $\hat\beta=0$.

Equating the right and left sides we get:

$$\frac{1+\hat\beta}{(1+\hat\beta)\widehat{P(A=1)}+\widehat{P(A=0)}}=1+\beta/2$$

which will be approximately true if $P(\widehat{A=1}) \sim P(\widehat{A=1|Y=0}) = 0.5$. That the approximation works best (in terms of ratio-bias) when the prevalence of the risk factor is roughly 0.5 is shown numerically in the Figure 3.

3 Proof of equivalence of (1) and (2) in the main manuscript.

Assume a continuous covariate C with possible values in the real line, and density f(c) with respect to Lebesgue measure. The argument below can be generalized for multidimensional C mixing both discrete and continuous variables by replacing the integrals with a mixture of integrals and summations over the respective ranges of the measured covariates.

$$\mathrm{PAF} = \frac{P(Y=1) - P(Y^{a=0}=1)}{P(Y=1)}$$

$$\frac{E(E(Y|A,C)) - E(E(Y^{a=0}|A,C))}{P(Y=1)}$$

$$= \frac{\sum_{a\in 0,1} \int P(Y=1|A=a,C=c) - P(Y^{a=0}=1|A=a,C=c)\, f(c)P(A=a|C=c)dc}{P(Y=1)}$$

Note that the conditional exchangeability condition is that $Y^{a=0}$ and A are independent given the covariates c. This implies that $P(Y^{a=0}=1|A=a,C=c) = P(Y^{a=0}=1|A=0,C=c)$. Noting that $Y = Y^{a=0}$ when A=0, it follows that $P(Y^{a=0}=1|A=0,C=c) = P(Y=1|A=0,C=c)$. Plugging this into the above:

$$= \frac{\sum_{a\in 0,1} \int (P(Y=1|A=a,C=c) - P(Y=1|A=0,C=c))\, P(A=a|C=c)f(c)dc}{P(Y=1)}$$

$$= \frac{\int (P(Y=1|A=1,C=c) - P(Y=1|A=0,C=c))\, P(A=1|C=c)f(c)dc}{P(Y=1)}$$

$$= \frac{\int (1-\mathrm{RR}^{-1})(P(Y=1|A=1,C=c)\, P(A=1|C=c)f(c)dc}{P(Y=1)}$$

$$= (1-\mathrm{RR}^{-1})\frac{\int (P(Y=1,A=1|C=c)f(c)dc}{P(Y=1)}$$

$$= (1-\mathrm{RR}^{-1})\frac{P(A=1,Y=1)}{P(Y=1)}$$

$$= (1-\mathrm{RR}^{-1})P(A=1|Y=1)$$
